# Supplementary material for: Complementary stable and dynamic prelimbic ensembles encode learned threat value underlying generalization and discrimination
Source: eLife. 2026 Oct 21;15:RP111177. doi: 10.7554/eLife.111177 (PMC13686620; doi:10.7554/eLife.111177)
Supplement: Supplementary file 2 [file elife-111177-supp1.docx]

**Supplementary File 1.**

**Fig. 2.**

| **Analysis** | **Cell class/session** | **Variable** | **Sum Sq.** | **Mean Sq.** | **F** | **df** | **p** | **ηp²** |
| --- | --- | --- | --- | --- | --- | --- | --- | --- |
| One-way ANOVA | Consistently active neurons | Group | 0.0261 | 0.0130 | 2.704 | 2, 13 | 0.104 | 0.29 |
| One-way ANOVA | Emerged-retained neurons: Day 1 and 15 | Group | 0.0065 | 0.00325 | 1.445 | 2, 13 | 0.271 | 0.18 |
| One-way ANOVA | Emerged-retained neurons: Day 15 and 30 | Group | 0.00697 | 0.00349 | 1.018 | 2, 13 | 0.388 | 0.14 |
| One-way ANOVA | Emerged-retained neurons: Day 1 and 30 | Group | 0.0084 | 0.0042 | 0.332 | 2, 13 | 0.723 | 0.05 |
| One-way ANOVA | Transiently active neurons: Day 1 | Group | 0.000194 | 0.000097 | 0.0598 | 2, 13 | 0.942 | 0.01 |
| Kruskal-Wallis | Transiently active neurons: Day 15 | Group | NA | NA | 1.901 | 2 | 0.387 | ε²=0.00 |
| Kruskal-Wallis | Transiently active neurons: Day 30 | Group | NA | NA | 7.515 | 2 | 0.023* | ε²=0.42 |

**Pairwise multiple comparisons (Dunn's method)**

| **Comparison** | **Diff. of ranks** | **Q** | **p** |
| --- | --- | --- | --- |
| No shock vs. CS+15 | 8.179 | 2.741 | 0.018* |
| No shock vs. CS+3 | 5.350 | 1.675 | 0.282 |
| CS+15 vs. CS+3 | 2.829 | 1.015 | 0.931 |

**Supplementary File 1 (Fig. 2).** Statistical analyses corresponding to the Venn diagrams shown in Fig. 2. One-way ANOVAs were used when the assumptions of normality and homogeneity of variance were met; otherwise, Kruskal–Wallis tests were performed. Significant Kruskal–Wallis tests were followed by Dunn's multiple-comparisons tests. Effect sizes are reported as partial eta squared (ηp²) for ANOVAs and epsilon squared (ε²) for Kruskal–Wallis tests.
