# Supplementary material for: Complementary stable and dynamic prelimbic ensembles encode learned threat value underlying generalization and discrimination
Source: eLife. 2026 Oct 21;15:RP111177. doi: 10.7554/eLife.111177 (PMC13686620; doi:10.7554/eLife.111177)
Supplement: Supplementary file 3 [file elife-111177-supp2.docx]

**Supplementary File 2**

**Fig. 3. 3-way mixed model ANOVA**

**a. Positive modulated**

| **Modulation** | **Variable** | **Sum Sq.** | **Mean Sq.** | **F** | **df** | **p** | **ηp²** |
| --- | --- | --- | --- | --- | --- | --- | --- |
| Positive | Group | 41614 | 20807.1 | 16.803 | 2, 17.907 | < 0.001*** | 0.65 |
|  | Time | 11118 | 5558.8 | 4.489 | 2, 191.314 | < 0.02* | 0.04 |
|  | Frequency | 3480 | 1160.1 | 0.937 | 3, 189.815 | 0.424 | 0.01 |
|  | Group x Time | 3617 | 904.3 | 0.730 | 4, 191.152 | 0.572 | 0.02 |
|  | Group x Frequency | 189326 | 31554.4 | 25.482 | 6, 189.815 | < 0.001*** | 0.45 |
|  | Time x Frequency | 9127 | 1521.1 | 1.228 | 6, 189.815 | 0.293 | 0.04 |
|  | Group x Time x Frequency | 7936 | 661.3 | 0.534 | 12, 189.815 | 0.891 | 0.03 |

**Simple effects analysis conducted for Group x Frequency interaction**

**Group = CS+15**

| **Contrast** | **Estimate** | **SE** | **df** | **t.ratio** | **p** |
| --- | --- | --- | --- | --- | --- |
| 3 vs 7 | -45.634 | 11.9 | 230 | -3.820 | < 0.0005*** |
| 3 vs 11 | -87.231 | 11.9 | 230 | -7.302 | < 0.0001*** |
| 3 vs 15 | -103.651 | 11.9 | 230 | -8.677 | < 0.0001*** |
| 7 vs 11 | -41.598 | 11.9 | 230 | -3.482 | < 0.0012** |
| 7 vs 15 | -58.018 | 11.9 | 230 | -4.857 | < 0.0001*** |
| 11 vs 15 | -16.420 | 11.9 | 230 | -1.375 | 0.1706 |

**Group = CS+3**

| **Contrast** | **Estimate** | **SE** | **df** | **t.ratio** | **p** |
| --- | --- | --- | --- | --- | --- |
| 3 vs 7 | 59.286 | 14.1 | 230 | 4.194 | < 0.0002*** |
| 3 vs 11 | 81.162 | 14.1 | 230 | 5.742 | < 0.0001*** |
| 3 vs 15 | 80.288 | 14.1 | 230 | 5.680 | < 0.0001*** |
| 7 vs 11 | 21.876 | 14.1 | 230 | 1.548 | 0.3692 |
| 7 vs 15 | 21.002 | 14.1 | 230 | 1.486 | 0.3692 |
| 11 vs 15 | -0.874 | 14.1 | 230 | -0.062 | 0.9508 |

**Group = No shock control**

| **Contrast** | **Estimate** | **SE** | **df** | **t.ratio** | **p** |
| --- | --- | --- | --- | --- | --- |
| 3 vs 7 | -15.841 | 13.9 | 230 | -1.137 | 1.0000 |
| 3 vs 11 | -10.626 | 13.9 | 230 | -0.762 | 1.0000 |
| 3 vs 15 | -7.660 | 13.9 | 230 | -0.550 | 1.0000 |
| 7 vs 11 | 5.216 | 13.9 | 230 | 0.374 | 1.0000 |
| 7 vs 15 | 8.181 | 13.9 | 230 | 0.587 | 1.0000 |
| 11 vs 15 | 2.966 | 13.9 | 230 | 0.213 | 1.0000 |

**b. Negative modulated**

| **Modulation** | **Variable** | **Sum Sq.** | **Mean Sq.** | **F** | **df** | **p** | **ηp²** |
| --- | --- | --- | --- | --- | --- | --- | --- |
| Negative | Group | 5701.6 | 2850.8 | 2.2670 | 2, 18.142 | 0.1322 | 0.200 |
|  | Time | 963.6 | 481.8 | 0.3831 | 2, 189.038 | 0.6822 | 0.004 |
|  | Frequency | 6148.4 | 2049.5 | 1.6298 | 3, 186.216 | 0.1840 | 0.030 |
|  | Group x Time | 1204.1 | 301.0 | 0.2394 | 4, 188.686 | 0.9158 | 0.005 |
|  | Group x Frequency | 14708.2 | 2451.4 | 1.9494 | 6, 186.203 | 0.0750 | 0.059 |
|  | Time x Frequency | 23070.6 | 3845.1 | 3.0577 | 6, 186.257 | < 0.0071** | 0.090 |
|  | Group x Time x Frequency | 23011.2 | 1917.6 | 1.5249 | 12, 186.238 | 0.1182 | 0.089 |

**Simple effect analysis conducted for Time x Frequency interaction**

**Time: Day 1**

| **Contrast** | **Estimate** | **SE** | **df** | **t.ratio** | **p** |
| --- | --- | --- | --- | --- | --- |
| 3 vs 7 | 14.787 | 13.2 | 226 | -1.124 | 1.0000 |
| 3 vs 11 | -15.219 | 13.7 | 227 | -1.109 | 1.0000 |
| 3 vs 15 | -8.945 | 13.2 | 226 | -0.680 | 1.0000 |
| 7 vs 11 | -0.433 | 13.7 | 227 | -0.032 | 1.0000 |
| 7 vs 15 | 5.842 | 13.2 | 226 | 0.444 | 1.0000 |
| 11 vs 15 | -6.274 | 13.7 | 227 | 0.457 | 1.0000 |

**Time: Day 15**

| **Contrast** | **Estimate** | **SE** | **df** | **t.ratio** | **p** |
| --- | --- | --- | --- | --- | --- |
| 3 vs 7 | -14.825 | 13.7 | 227 | -1.080 | 1.0000 |
| 3 vs 11 | 9.416 | 13.2 | 226 | 0.716 | 1.0000 |
| 3 vs 15 | 3.458 | 13.2 | 226 | 0.263 | 1.0000 |
| 7 vs 11 | 24.241 | 13.7 | 227 | 1.766 | 0.4726 |
| 7 vs 15 | 18.283 | 13.7 | 227 | 1.332 | 0.9212 |
| 11 vs 15 | 5.958 | 13.2 | 226 | -0.453 | 1.0000 |

**Time: Day 30**

| **Contrast** | **Estimate** | **SE** | **df** | **t.ratio** | **p** |
| --- | --- | --- | --- | --- | --- |
| 3 vs 7 | -15.841 | 13.9 | 230 | -1.137 | 1.0000 |
| 3 vs 11 | -10.626 | 13.9 | 230 | -0.762 | 1.0000 |
| 3 vs 15 | -7.660 | 13.9 | 230 | -0.550 | 1.0000 |
| 7 vs 11 | 5.216 | 13.9 | 230 | 0.374 | 1.0000 |
| 7 vs 15 | 8.181 | 13.9 | 230 | 0.587 | 1.0000 |
| 11 vs 15 | 2.966 | 13.9 | 230 | 0.213 | 1.0000 |

**Supplementary File 2 (Fig. 3). Statistics comparing areas under the curve for the CS+15, CS+3, and No shock control groups across all frequencies and testing sessions.** (a-b) Three-way mixed model ANOVAs using Group (CS+15, CS+3, No shock), Frequency (3, 7, 11, 15 kHz), and Time (days 1, 15, 30) as variables for positive (a) and negative (b) cell responders. Simple effects were calculated using Holm correction.
