# Supplementary material for: Complementary stable and dynamic prelimbic ensembles encode learned threat value underlying generalization and discrimination
Source: eLife. 2026 Oct 21;15:RP111177. doi: 10.7554/eLife.111177 (PMC13686620; doi:10.7554/eLife.111177)
Supplement: Supplementary file 4 [file elife-111177-supp3.docx]

| **Group** | **Type** | **Modulation** | **Test** | **Statistic** | **df** | **p** η**p^2^** |
| --- | --- | --- | --- | --- | --- | --- |
| CS+15 | Consistent | Positive | Test Day 1 | F = 6.11 | 3, 18 | 0.005 ** 0.51 |
|  |  |  | Test Day 15 | F = 6.29 | 3, 18 | 0.004** 0.51 |
|  |  |  | Test Day 30 | F = 4.43 | 3, 18 | 0.017* 0.43 |
|  |  | Negative | Test Day 1 | F = 3.76 | 3, 18 | 0.030* 0.39 |
|  |  |  | Test Day 15 | F = 0.31 | 3, 18 | 0.817 0.05 |
|  |  |  | Test Day 30 | F = 1.80 | 3, 18 | 0.183 0.23 |
|  | Emerged Retained | Positive | Test Day 1 | F = 3.18 | 3, 13 | 0.060 0.42 |
|  |  |  | Test Day 15 | F = 3.24 | 3, 18 | 0.046 * 0.35 |
|  |  |  | Test Day 30 | χ² = 8.66 | 3 | 0.0.4* NA |
|  |  | Negative | Test Day 1 | F = 1.89 | 3, 17 | 0.170 0.25 |
|  |  |  | Test Day 15 | F = 1.45 | 3, 18 | 0.263 0.20 |
|  |  |  | Test Day 30 | F = 2.63 | 3, 18 | 0.308 0.31 |
|  | Transiently Active | Positive | Test Day 1 | F = 1.18 | 3, 17 | 0.332 0.17 |
|  |  |  | Test Day 15 | F = 11.17 | 3, 18 | <0.001***0.65 |
|  |  |  | Test Day 30 | F = 3.87 | 3, 18 | 0.030 * 0.39 |
|  |  | Negative | Test Day 1 | F = 0.39 | 3, 17 | 0.764 0.06 |
|  |  |  | Test Day 15 | F = 0.30 | 3, 18 | 0.827 0.05 |
|  |  |  | Test Day 30 | F = 2.08 | 3, 18 | 0.139 0.26 |
| CS+3 | Consistent | Positive | Test Day 1 | F = 9.32 | 3, 12 | 0.002 ** 0.70 |
|  |  |  | Test Day 15 | F = 3.18 | 3, 12 | 0.063 0.44 |
|  |  |  | Test Day 30 | F = 5.39 | 3, 12 | 0.014 * 0.57 |
|  |  | Negative | Test Day 1 | F = 1.28 | 3, 12 | 0.326 0.24 |
|  |  |  | Test Day 15 | F = 0.53 | 3, 12 | 0.668 0.12 |
|  |  |  | Test Day 30 | F = 0.47 | 3, 12 | 0.709 0.11 |
|  | Emerged Retained | Positive | Test Day 1 | F = 1.49 | 3, 6 | 0.309 0.43 |
|  |  |  | Test Day 15 | F = 6.18 | 3, 9 | 0.014 * 0.67 |
|  |  |  | Test Day 30 | F = 9.51 | 3, 9 | 0.004 ** 0.76 |
|  |  | Negative | Test Day 1 | F = 4.75 | 3, 8 | 0.035 0.64 |
|  |  |  | Test Day 15 | F = 0.65 | 3, 12 | 0.595 0.14 |
|  |  |  | Test Day 30 | χ² = 1.39 | 3 | 0.356 NA |
|  | Transiently Active | Positive | Test Day 1 | F = 0.28 | 3, 8 | 0.839 0.10 |
|  |  |  | Test Day 15 | F = 6.45 | 3, 12 | 0.008 **0.62 |
|  |  |  | Test Day 30 | F = 5.49 | 3, 12 | 0.013 * 0.58 |
|  |  | Negative | Test Day 1 | F = 1.12 | 3, 9 | 0.398 0.27 |
|  |  |  | Test Day 15 | F = 1.57 | 3, 11 | 0.253 0.30 |
|  |  |  | Test Day 30 | F = 0.426 | 3, 12 | 0.738 0.10 |

**Supplementary File 3**

**Fig. S4, S5, S6**

**Supplementary File 3. Statistics corresponding to consistently active, emerging-retained, and transiently active cells.** One-way ANOVAs with repeated measures were used when normality and equal variance tests were passed to test the effect of frequency. Friedman tests were used (χ² with Kendall’s W) when assumptions were violated, Friedman tests were used (χ² with Kendall’s W).
