# Supplementary material for: Complementary stable and dynamic prelimbic ensembles encode learned threat value underlying generalization and discrimination
Source: eLife. 2026 Oct 21;15:RP111177. doi: 10.7554/eLife.111177 (PMC13686620; doi:10.7554/eLife.111177)
Supplement: Supplementary file 5 [file elife-111177-supp4.docx]

**Supplementary File 4**

**Fig. 4**

**Positive modulated cells**

**Fig. 4a**

| **Modulation** | **Variable** | **Sum Sq.** | **Mean Sq.** | **F** | **df** | **p** | **ηp²** |
| --- | --- | --- | --- | --- | --- | --- | --- |
| Positive | Group | 8959 | 4.48 | 9.14 | 2, 15 | 0.003** | 0.55 |
|  | Frequency | 7451 | 0.49 | 2.12 | 3, 45 | 0.111 | 0.12 |
|  | Group x Frequency | 4859 | 0.81 | 8.69 | 6, 45 | < 0.001*** | 0.54 |

**Tukey post hoc comparisons**

| **Tone** | **Comparison** | **Diff. of means** | **df** | **q** | **p** |
| --- | --- | --- | --- | --- | --- |
| 3 kHz | CS+3 vs. No shock | 0.747 | 3 | 3.975 | 0.022* |
|  | CS+3 vs. CS+15 | 0.491 | 3 | 2.706 | 0.151 |
|  | CS+15 vs. No shock | 0.255 | 3 | 1.479 | 0.554 |
| 7 kHz | CS+15 vs. No shock | 0.684 | 3 | 3.963 | 0.022* |
|  | CS+15 vs. CS+3 | 0.229 | 3 | 1.259 | 0.650 |
|  | CS+3 vs. No shock | 0.455 | 3 | 2.423 | 0.215 |
| 11 kHz | CS+15 vs. No shock | 1.174 | 3 | 6.805 | < 0.001*** |
|  | CS+15 vs. CS+3 | 0.737 | 3 | 4.058 | 0.019* |
|  | CS+3 vs. No shock | 0.437 | 3 | 2.328 | 0.241 |
| 15 kHz | CS+15 vs. No shock | 1.215 | 3 | 7.040 | < 0.001*** |
|  | CS+15 vs. CS+3 | 1.176 | 3 | 6.476 | < 0.001*** |
|  | CS+3 vs. No shock | 0.0387 | 3 | 0.206 | 0.988 |

**Fig. 4b**

| **Modulation** | **Variable** | **Sum Sq.** | **Mean Sq.** | **F** | **df** | **p** | **ηp²** |
| --- | --- | --- | --- | --- | --- | --- | --- |
| positive | Group | 0.26 | 0.13 | 2.79 | 2, 15 | 0.094 | 0.27 |
|  | Day | 0.02 | 0.007 | 0.806 | 3, 45 | 0.497 | 0.05 |
|  | Group x Day | 0.154 | 0.025 | 2.84 | 6, 45 | 0.020* | 0.27 |

**Tukey post hoc comparisons**

| **Tone** | **Comparison** | **Diff. of means** | **df** | **q** | **p** |
| --- | --- | --- | --- | --- | --- |
| 3 kHz | No shock vs. CS+3 | 0.164 | 3 | 2.818 | 0.130 |
|  | No shock vs. CS+15 | 0.0454 | 3 | 0.849 | 0.821 |
|  | CS+15 vs. CS+3 | 0.119 | 3 | 2.107 | 0.308 |
| 7 kHz | No shock vs. CS+15 | 0.166 | 3 | 3.099 | 0.087 |
|  | No shock vs. CS+3 | 0.105 | 3 | 1.800 | 0.420 |
|  | CS+3 vs. CS+15 | 0.061 | 3 | 1.084 | 0.726 |
| 11 kHz | No shock vs. CS+15 | 0.185 | 3 | 3.455 | 0.051 |
|  | No shock vs. CS+3 | 0.0418 | 3 | 0.718 | 0.868 |
|  | CS+3 vs. CS+15 | 0.143 | 3 | 2.539 | 0.186 |
| 15 kHz | No shock vs. CS+15 | 0.172 | 3 | 3.207 | 0.074 |
|  | No shock vs. CS+3 | 0.000427 | 3 | 0.00734 | 1.000 |
|  | CS+3 vs. CS+15 | 0.171 | 3 | 3.040 | 0.095 |

**Fig. 4c**

| **Modulation** | **Variable** | **Sum Sq.** | **Mean Sq.** | **F** | **df** | **p** | **ηp²** |
| --- | --- | --- | --- | --- | --- | --- | --- |
| Positive | Group | 0.400 | 0.200 | 3.802 | 2, 15 | 0.046* | 0.336 |
|  | Condition | 3.983 | 3.983 | 176.813 | 1, 15 | < 0.001*** | 0.922 |
|  | Group x Condition | 0.267 | 0.133 | 5.921 | 2, 15 | 0.013* | 0.441 |

**Tukey post hoc comparisons**

| **Factor** | **Comparison** | **Diff. of means** | **df** | **q** | **p** |
| --- | --- | --- | --- | --- | --- |
| within CS+15 | Tone vs. freezing | 0.862 | 2 | 15.238 | < 0.001*** |
| within CS+3 | Tone vs. freezing | 0.696 | 2 | 10.361 | < 0.001*** |
| within Nonshock | Tone vs. freezing | 0.457 | 2 | 7.429 | < 0.001*** |
| within freezing | CS+15 vs. Nonshock | 0.045 | 3 | 0.684 | 0.880 |
|  | CS+15 vs. CS+3 | 0.054 | 3 | 0.779 | 0.848 |
|  | Nonshock vs. CS+3 | 0.009 | 3 | 0.125 | 0.996 |
| within tone | CS+15 vs. Nonshock | 0.451 | 3 | 5.271 | 0.005** |
|  | CS+15 vs. CS+3 | 0.221 | 3 | 2.461 | 0.223 |
|  | CS+3 vs. Nonshock | 0.230 | 3 | 2.483 | 0.218 |

**Fig. 4d**

| **Modulation** | **Variable** | **Sum Sq.** | **Mean Sq.** | **F** | **df** | **p** | **ηp²** |
| --- | --- | --- | --- | --- | --- | --- | --- |
| Positive | Group | 156.357 | 78.178 | 1.166 | 2, 15 | 0.336 | 0.13 |
|  | Day | 17.999 | 68.24 | 0.174 | 2, 26 | 0.842 | 0.01 |
|  | Group x Day | 106.236 | 26.559 | 0.512 | 4, 26 | 0.727 | 0.07 |

**Negative modulated cells**

**Fig. 4e**

| **Modulation** | **Variable** | **Sum Sq.** | **Mean Sq.** | **F** | **df** | **p** | **ηp²** |
| --- | --- | --- | --- | --- | --- | --- | --- |
| Negative | Group | 0.135 | 0.0677 | 0.968 | 2, 15 | 0.402 | 0.11 |
|  | Frequency | 0.101 | 0.0336 | 1.453 | 3, 44 | 0.240 | 0.09 |
|  | Group x Frequency | 0.072 | 0.012 | 0.519 | 6, 44 | 0.791 | 0.07 |

**Fig. 4f**

| **Modulation** | **Variable** | **Sum Sq.** | **Mean Sq.** | **F** | **df** | **p** | **ηp²** |
| --- | --- | --- | --- | --- | --- | --- | --- |
| Negative | Group | 1.038 | 0.519 | 3.066 | 2, 15 | 0.076 | 0.29 |
|  | Frequency | 0.141 | 0.0471 | 0.399 | 3, 44 | 0.754 | 0.03 |
|  | Group x Frequency | 0.468 | 0.0779 | 0.661 | 6, 44 | 0.681 | 0.08 |

**Fig. 4g**

| **Modulation** | **Variable** | **Sum Sq.** | **Mean Sq.** | **F** | **df** | **p** | **ηp²** |
| --- | --- | --- | --- | --- | --- | --- | --- |
| Negative | Group | 0.004 | 0.002 | 0.061 | 2, 15 | 0.941 | 0.008 |
|  | Condition | 0.308 | 0.308 | 32.496 | 1, 15 | < 0.001*** | 0.684 |
|  | Group x Condition | 0.101 | 0.050 | 5.315 | 2, 15 | 0.018* | 0.415 |

**Tukey post hoc comparisons**

| **Factor** | **Comparison** | **Diff. of means** | **df** | **q** | **p** |
| --- | --- | --- | --- | --- | --- |
| within CS+15 | Tone vs. freezing | 0.302 | 2 | 8.213 | < 0.001*** |
| within CS+3 | Tone vs. freezing | 0.206 | 2 | 4.699 | 0.005** |
| within Nonshock | Tone vs. freezing | 0.053 | 2 | 1.338 | 0.359 |
| within freezing | Nonshock vs. CS+15 | 0.100 | 3 | 1.473 | 0.563 |
|  | Nonshock vs. CS+3 | 0.070 | 3 | 0.943 | 0.786 |
|  | CS+3 vs. CS+15 | 0.030 | 3 | 0.420 | 0.953 |
| within tone | CS+15 vs. Nonshock | 0.149 | 3 | 3.145 | 0.099 |
|  | CS+15 vs. CS+3 | 0.066 | 3 | 1.315 | 0.631 |
|  | CS+3 vs. Nonshock | 0.083 | 3 | 1.608 | 0.507 |

**Fig. 4h**

| **Modulation** | **Variable** | **Sum Sq.** | **Mean Sq.** | **F** | **df** | **p** | **ηp²** |
| --- | --- | --- | --- | --- | --- | --- | --- |
| Negative | Group | 886.884 | 443.442 | 2.756 | 2, 15 | 0.092 | 0.27 |
|  | Day | 400.272 | 200.136 | 1.500 | 2, 26 | 0.242 | 0.10 |
|  | Group x Day | 468.189 | 117.047 | 0.877 | 4, 26 | 0.491 | 0.12 |

**Supplementary File 4 (Fig. 4).** Statistical analysis of GLM-derived measures for cells used in the population curves shown in Fig. 4. Two-way ANOVAs with repeated measures were conducted to evaluate effects of group (CS+15, CS+3, or No shock), frequency (3, 7, 11, 15 kHz) or day (days 1, 15, 30), and their interactions. Tukey post hoc comparisons are shown where simple effects were tested.
