# Supplementary material for: Complementary stable and dynamic prelimbic ensembles encode learned threat value underlying generalization and discrimination
Source: eLife. 2026 Oct 21;15:RP111177. doi: 10.7554/eLife.111177 (PMC13686620; doi:10.7554/eLife.111177)
Supplement: Supplementary file 6 [file elife-111177-supp5.docx]

| **Supplementary File 5**  **Fig. 6 and 7** |  | |  | | |  | | |  | | |  | | |  | | |  | | |
| --- | --- | --- | --- | --- | --- | --- | --- | --- | --- | --- | --- | --- | --- | --- | --- | --- | --- | --- | --- | --- |
| **Group** | **Cluster Mod.** | **% Stable D1-D15** | | **P (D1-D15)** | | | **% Stable D15-D30** | | | **P (D15-D30)** | | | **% Stable D1-D30** | | | **P (D1-D30)** | | |  |  |
| CS+15  Fig. 6  Fig. 7 | Single tone c1 + | 13.5 | | | 0.391 | | | 5.0 | | | 1.000 | | | 9.3 | | | 0.870 | | |  |
|  | Single tone c2 + | 3.4 | | | 1.000 | | | 7.1 | | | 1.000 | | | 8.2 | | | 1.000 | | |  |
|  | Single tone c3 + | 11.1 | | | 0.576 | | | 13.4 | | | 0.391 | | | 19.2 | | | 0.124 | | |  |
|  | Single tone c4 + | 15.5 | | | 0.391 | | | 15.1 | | | 0.391 | | | 20.9 | | | 0.121 | | |  |
|  | Single tone d1 - | 19.3 | | | 0.079 | | | 11.9 | | | 0.391 | | | 12.9 | | | 0.576 | | |  |
|  | Single tone d2 - | 10.4 | | | 0.765 | | | 10.0 | | | 0.661 | | | 10.3 | | | 0.576 | | |  |
|  | Single tone d3 - | 11.9 | | | 0.697 | | | 20.2 | | | 0.334 | | | 17.8 | | | 0.697 | | |  |
|  | Single tone d4 - | 20.2 | | | 0.011* | | | 11.8 | | | 1.000 | | | 10.3 | | | 1.000 | | |  |
|  | Graded a | 28.2 | | | 0.000*** | | | 22.8 | | | 0.000*** | | | 21.3 | | | 0.001*** | | |  |
|  | Graded b | 11.1 | | | 0.034* | | | 5.5 | | | 0.576 | | | 5.9 | | | 0.576 | | |  |
| CS+3  Fig. 6  Fig. 7 | Single tone c1 + | 23.9 | | | 0.014* | | | 16.5 | | | 0.460 | | | 19.0 | | | 0.476 | | |  |
|  | Single tone c2 + | 13.4 | | | 0.708 | | | 6.5 | | | 0.982 | | | 11.9 | | | 0.792 | | |  |
|  | Single tone c3 + | 10.8 | | | 0.852 | | | 9.8 | | | 0.939 | | | 21.1 | | | 0.185 | | |  |
|  | Single tone c4 + | 10.4 | | | 0.756 | | | 7.7 | | | 0.982 | | | 19.6 | | | 0.521 | | |  |
|  | Single tone d1 - | 20.9 | | | 0.230 | | | 20.6 | | | 0.074 | | | 13.0 | | | 0.708 | | |  |
|  | Single tone d2 - | 11.5 | | | 0.714 | | | 8.0 | | | 0.579 | | | 8.6 | | | 0.592 | | |  |
|  | Single tone d3 - | 9.7 | | | 0.739 | | | 5.3 | | | 0.982 | | | 10.3 | | | 0.846 | | |  |
|  | Single tone d4 - | 8.9 | | | 0.872 | | | 7.6 | | | 0.972 | | | 12.8 | | | 0.592 | | |  |
|  | Graded c | 21.2 | | | 0.008** | | | 17.9 | | | 0.026* | | | 25.0 | | | 0.008** | | |  |
|  | Graded d | 9.1 | | | 0.303 | | | 40.0 | | | 0.003** | | | 14.3 | | | 0.303 | | |  |
|  | Graded e | 12.9 | | | 0.047* | | | 15.4 | | | 0.047* | | | 8.3 | | | 0.589 | | |  |
| No Shock  Fig. 6 | Single tone c1 + | 7.8 | | | 1.000 | | | 8.3 | | | 1.000 | | | 22.2 | | | 1.000 | | |  |
|  | Single tone c2 + | 11.5 | | | 1.000 | | | 10.0 | | | 1.000 | | | 0.0 | | | 1.000 | | |  |
|  | Single tone c3 + | 21.6 | | | 1.000 | | | 16.7 | | | 1.000 | | | 40.0 | | | 1.000 | | |  |
|  | Single tone c4 + | 11.5 | | | 1.000 | | | 5.9 | | | 1.000 | | | 0.0 | | | 1.000 | | |  |
|  | Single tone d1 - | 10.0 | | | 1.000 | | | 7.1 | | | 1.000 | | | 0.0 | | | 1.000 | | |  |
|  | Single tone d2 - | 5.5 | | | 1.000 | | | 21.4 | | | 1.000 | | | 21.4 | | | 1.000 | | |  |
|  | Single tone d3 - | 12.0 | | | 1.000 | | | 4.0 | | | 1.000 | | | 33.3 | | | 1.000 | | |  |
|  | Single tone d4 - | 6.4 | | | 1.000 | | | 22.2 | | | 1.000 | | | 0.0 | | | 1.000 | | |  |

**Supplementary file 5 (Fig. 6 and 7).** Statistical analyses identifying neuronal clusters that remained stable over time, defined as neurons that retained the same functional response profile on days 15 and 30 as on day 1. These analyses correspond to the clusters shown in Figs. 6 and 7, as indicated in the table. For each cluster, the observed proportion of neurons that preserved their response profile was compared with a null distribution, with multiple comparisons controlled using the Benjamini–Hochberg procedure. Only the graded neuronal clusters in each experimental group (Fig. 7) exhibited significant stability across time. No graded clusters were identified in the no-shock control group.
