# Supplementary material for: Complementary stable and dynamic prelimbic ensembles encode learned threat value underlying generalization and discrimination
Source: eLife. 2026 Oct 21;15:RP111177. doi: 10.7554/eLife.111177 (PMC13686620; doi:10.7554/eLife.111177)
Supplement: Supplementary file 7 [file elife-111177-supp6.docx]

**Supplementary file 6**

**Fig. S10.**

| **Cluster** | **Groups** | **Day** | **Statistic** | **df** | **p** η**p^2^** |
| --- | --- | --- | --- | --- | --- |
| **Statistics for clusters**  **responding to individual frequencies (Fig. 6c)** | | | | | |
| Fig. 6c, c.1  Fig. S7, S8, S9  Maximal response  at 3 kHz | CS+15  CS+3  No shock | Test Day 1 | Group: F =1.49  Frequency: F=30.51  Interaction: 4.50 | 2, 15  3, 6  6, 45 | 0.258 0.17  0.001***. 0.94  0.001***. 0.38 |
|  |  | Test Day 15 | Group: F =4.59  Frequency: F=31.99  Interaction: 2.28 | 2, 15  3, 6  6,4 5 | 0.03* 0.38  0.001***. 0.94  0.053. 0.23 |
|  |  | Test Day 30 | Group: F =0.36  Frequency: F=30.02  Interaction: 2.78 | 3, 13  3, 6  6, 39 | 0.705 0.08  0.001***. 0.94  0.024*. 0.30 |
| Fig. 6c, c.2  Fig. S7, S8, S9  Maximal response  at 7kHz | CS+15  CS+3  No shock | Test Day 1 | Group: F =2.29  Frequency: F=30.18  Interaction: 2.62 | 2,15  3, 6  6, 45 | 0.135 0.23  0.001***. 0.94  0.029*. 0.26 |
|  |  | Test Day 15 | Group: F =3.50  Frequency: F=27.46  Interaction: 0.99 | 2, 15  3, 6  6, 45 | 0.057 0.32  0.001***. 0.93  0.441. 0.12 |
|  |  | Test Day 30 | Group: F =3.51  Frequency: F=24.64  Interaction: 1.057 | 2,13  3, 6  6, 3 9 | 0.061 0.35  0.001***. 0.93  0.404. 0.14 |
| Fig. 6c, c.3  Fig. S7, S8, S9  Maximal response  at 11 kHz | CS+15  CS+3  No shock | Test Day 1 | Group: F =1.45  Frequency: F=20.44  Interaction: 0.912 | 2, 15  3, 6  6, 4 5 | 0.266 0.16  0.001***. 0.91  0.495. 0.11 |
|  |  | Test Day 15 | Group: F =15.47  Frequency: F=38.53  Interaction: 3.40 | 2, 15  3, 6  6,4 5 | 0.001*** 0.67  0.001***. 0.95  0.007**. 0.31 |
|  |  | Test Day 30 | Group: F =11.46  Frequency: F=14.20  Interaction: 6.12 | 2, 13  3, 6  6, 3 9 | 0.001*** 0.64  0.001***. 0.88  0.001***. 0.49 |
| Fig. 6c, c.4  Fig. S7, S8, S9  Maximal response  at 15 kHz | CS+15  CS+3  No shock | Test Day 1 | Group: F =2.71  Frequency: F=22.28  Interaction: 0.38 | 3, 15  3, 6  6,4 5 | 0.099 0.35  0.001***. 0.92  0.89. 0.05 |
|  |  | Test Day 15 | Group: F =7.64  Frequency: F=33.86  Interaction: 10.58 | 2,1 5  3, 6  6, 45 | 0.005** 0.51  0.001***. 0.94  0.001***. 0.59 |
|  |  | Test Day 30 | Group: F =8.01  Frequency: F=30.05  Interaction: 4.15 | 2, 13  3, 6  6, 39 | 0.005** 0.55  0.001***. 0.94  0.003**. 0.39 |
| **Statistics for clusters showing graded valence (Fig.7)** | | | | | |
| Graded cluster  Fig. 7a  Fig. S7a | Experimental CS+15 | Test Day 1, 15, and 30 | Day: F =2.89  Frequency: F=29.28  Interaction: 0.351 | 2, 12  3, 12  6,36 | 0.10 0.33  0.001***. 0.88  0.905. 0.06 |
| Graded cluster  Fig. 7c  Fig. S8a | Experimental CS+3 | Test Day 1,  15, and 30 | Day F =0.467  Frequency: F=53.37  Interaction: 2.63 | 2, 8  3,12  6, 24 | 0.643 0.11  0.001***. 0.93  0.042*. 0.40 |

**Supplementary File 6 (Fig. S10).** Statistical analyses corresponding to the normalized baseline-to-stimulus firing rate (BSR) for the neuronal clusters shown in Figs. S7–S9. BSRs for the clusters shown in Fig. S7-S9 are presented in Fig. S10, whereas BSRs for the graded clusters shown in Figs. S7-S8 are presented in Figs. 7h–i. For pure tone-selective clusters, two-way repeated-measures ANOVAs were used to evaluate the effects of group (CS+15, CS+3, or No Shock), frequency (3, 7, 11, and 15 kHz), and their interaction. For graded clusters, one-way repeated-measures ANOVAs were used to evaluate the effect of time. Tukey's multiple-comparisons tests are reported in Figs. S10 and 7h–i.
