# Supplementary material for: Complementary stable and dynamic prelimbic ensembles encode learned threat value underlying generalization and discrimination
Source: eLife. 2026 Oct 21;15:RP111177. doi: 10.7554/eLife.111177 (PMC13686620; doi:10.7554/eLife.111177)
Supplement: Supplementary file 8 [file elife-111177-supp7.docx]

**Supplementary File 7**

**Fig. 8**

**Positive modulated single-frequency neurons**

**Fig. 8a**

| **Modulation** | **Variable** | **Sum Sq.** | **Mean Sq.** | **F** | **df** | **p** | **ηp²** |
| --- | --- | --- | --- | --- | --- | --- | --- |
| Positive | Group | 15.905 | 7.952 | 19.447 | 2, 15 | < 0.001*** | 0.72 |
|  | Frequency | 0.832 | 0.277 | 1.108 | 3, 45 | 0.356 | 0.07 |
|  | Group x Frequency | 6.319 | 1.053 | 4.21 | 6, 45 | 0.002** | 0.36 |

**Tukey post hoc comparisons**

| **Tone** | **Comparison** | **Diff. of means** | **df** | **q** | **p** |
| --- | --- | --- | --- | --- | --- |
| 3 kHz | CS+3 vs. No shock | 0.757 | 3 | 3.284 | 0.061 |
|  | CS+3 vs. CS+15 | 0.374 | 3 | 1.677 | 0.467 |
|  | CS+15 vs. No shock | 0.383 | 3 | 1.810 | 0.412 |
| 7 kHz | CS+15 vs. No shock | 0.969 | 3 | 4.574 | < 0.006** |
|  | CS+15 vs. CS+3 | 0.341 | 3 | 1.530 | 0.529 |
|  | CS+3 vs. No shock | 0.628 | 3 | 2.723 | 0.141 |
| 11 kHz | CS+15 vs. No shock | 1.609 | 3 | 7.597 | < 0.001*** |
|  | CS+15 vs. CS+3 | 1.363 | 3 | 6.116 | < 0.001*** |
|  | CS+3 vs. No shock | 0.246 | 3 | 1.065 | 0.733 |
| 15 kHz | CS+15 vs. No shock | 1.443 | 3 | 6.811 | < 0.001*** |
|  | CS+15 vs. CS+3 | 1.218 | 3 | 5.464 | < 0.001*** |
|  | CS+3 vs. No shock | 0.225 | 3 | 0.975 | 0.771 |

**Fig. 8b**

| **Modulation** | **Variable** | **Sum Sq.** | **Mean Sq.** | **F** | **df** | **p** | **ηp²** |
| --- | --- | --- | --- | --- | --- | --- | --- |
| Positive | Group | 0.207 | 0.104 | 6.62 | 2, 15 | 0.008** | 0.47 |
|  | Day | 0.0178 | 0.0089 | 1.205 | 2, 26 | 0.316 | 0.08 |
|  | Group x Day | 0.0379 | 0.00948 | 1.281 | 4, 26 | 0.303 | 0.16 |

**Tukey post hoc comparisons**

| **Comparison** | **Diff. of means** | **df** | **q** | **p** |
| --- | --- | --- | --- | --- |
| No shock vs. CS+15 | 0.168 | 3 | 5.026 | < 0.008** |
| No shock vs. CS+3 | 0.117 | 3 | 3.316 | 0.080 |
| CS+3 vs. CS+15 | 0.0513 | 3 | 1.654 | 0.488 |

**Fig. 8c**

| **Modulation** | **Variable** | **Sum Sq.** | **Mean Sq.** | **F** | **df** | **p** | **ηp²** |
| --- | --- | --- | --- | --- | --- | --- | --- |
| Positive | Group | 0.49 | 0.245 | 5.277 | 2, 15 | 0.018* | 0.41 |
|  | Condition | 3.962 | 3.962 | 254.641 | 1, 15 | < 0.001*** | 0.94 |
|  | Group x Condition | 0.318 | 0.159 | 10.205 | 2, 15 | 0.002** | 0.58 |

**Tukey post hoc comparisons**

| **Factor** | **Comparison** | **Diff. of means** | **df** | **q** | **p** |
| --- | --- | --- | --- | --- | --- |
| within CS+15 | Tone vs. freezing | 0.899 | 2 | 19.078 | < 0.001*** |
| within CS+3 | Tone vs. freezing | 0.651 | 2 | 11.676 | < 0.001*** |
| within No shock | Tone vs. freezing | 0.459 | 2 | 9.005 | < 0.001*** |
| within freezing | CS+15 vs. No shock | 0.0531 | 3 | 0.767 | 0.851 |
|  | CS+15 vs. CS+3 | 0.0317 | 3 | 0.435 | 0.949 |
|  | CS+3 vs. No shock | 0.0214 | 3 | 0.284 | 0.978 |
| within tone | CS+15 vs. No shock | 0.494 | 3 | 7.134 | < 0.001*** |
|  | CS+15 vs. CS+3 | 0.280 | 3 | 3.839 | 0.031* |
|  | CS+3 vs. No shock | 0.214 | 3 | 2.843 | 0.131 |

**Fig. 8d**

| **Modulation** | **Variable** | **Sum Sq.** | **Mean Sq.** | **F** | **df** | **p** | **ηp²** |
| --- | --- | --- | --- | --- | --- | --- | --- |
| Positive | Group | 83.099 | 41.549 | 0.763 | 2, 15 | 0.482 | 0.09 |
|  | Day | 62.468 | 31.234 | 0.649 | 2, 26 | 0.531 | 0.05 |
|  | Group x Day | 130.193 | 32.548 | 0.677 | 4, 26 | 0.614 | 0.09 |

**Negative modulated single-frequency neurons**

**Fig. 8e**

| **Modulation** | **Variable** | **Sum Sq.** | **Mean Sq.** | **F** | **df** | **p** | **ηp²** |
| --- | --- | --- | --- | --- | --- | --- | --- |
| Negative | Group | 1.765 | 0.882 | 11.246 | 2, 15 | 0.001*** | 0.60 |
|  | Frequency | 0.121 | 0.0404 | 1.274 | 3, 45 | 0.295 | 0.08 |
|  | Group x Frequency | 0.166 | 0.0277 | 0.872 | 6, 45 | 0.523 | 0.10 |

**Tukey post hoc comparisons**

| **Comparison** | **Diff. of means** | **df** | **q** | **p** |
| --- | --- | --- | --- | --- |
| No shock vs. CS+15 | 0.365 | 3 | 6.624 | < 0.001*** |
| No shock vs. CS+3 | 0.142 | 3 | 2.361 | 0.249 |
| CS+3 vs. CS+15 | 0.223 | 3 | 3.852 | 0.039* |

**Fig. 8f**

| **Modulation** | **Variable** | **Sum Sq.** | **Mean Sq.** | **F** | **df** | **p** | **ηp²** |
| --- | --- | --- | --- | --- | --- | --- | --- |
| Negative | Group | 1.474 | 0.737 | 18.566 | 2, 15 | < 0.001*** | 0.71 |
|  | Frequency | 0.131 | 0.0656 | 1.509 | 2, 26 | 0.240 | 0.10 |
|  | Group x Frequency | 0.185 | 0.0462 | 1.062 | 4, 26 | 0.395 | 0.14 |

**Tukey post hoc comparisons**

| **Comparison** | **Diff. of means** | **df** | **q** | **p** |
| --- | --- | --- | --- | --- |
| No shock vs. CS+15 | 0.451 | 3 | 8.650 | < 0.001*** |
| No shock vs. CS+3 | 0.284 | 3 | 5.176 | 0.006** |
| CS+3 vs. CS+15 | 0.167 | 3 | 3.450 | 0.067 |

**Fig. 8g**

| **Modulation** | **Variable** | **Sum Sq.** | **Mean Sq.** | **F** | **df** | **p** | **ηp²** |
| --- | --- | --- | --- | --- | --- | --- | --- |
| Negative | Group | 0.149 | 0.0746 | 4.47 | 2, 15 | 0.030* | 0.37 |
|  | Day | 0.261 | 0.261 | 39.684 | 1, 15 | < 0.001*** | 0.73 |
|  | Group x Day | 0.174 | 0.087 | 13.233 | 2, 15 | < 0.001*** | 0.64 |

**Tukey post hoc comparisons**

| **Factor** | **Comparison** | **Diff. of means** | **df** | **q** | **p** |
| --- | --- | --- | --- | --- | --- |
| within CS+15 | Tone vs. freezing | 0.343 | 2 | 11.207 | < 0.001*** |
| within CS+3 | Tone vs. freezing | 0.154 | 2 | 4.257 | < 0.009** |
| within No shock | Tone vs. freezing | 0.0178 | 2 | 0.538 | 0.709 |
| within freezing | No shock vs. CS+15 | 0.0125 | 3 | 0.294 | 0.977 |
|  | No shock vs. CS+3 | 0.00849 | 3 | 0.184 | 0.991 |
|  | CS+3 vs. CS+15 | 0.00397 | 3 | 0.0889 | 0.998 |
| within tone | CS+15 vs. No shock | 0.313 | 3 | 7.379 | < 0.001*** |
|  | CS+15 vs. CS+3 | 0.185 | 3 | 4.144 | 0.019* |
|  | CS+3 vs. No shock | 0.128 | 3 | 2.772 | 0.143 |

**Fig. 8h**

| **Modulation** | **Variable** | **Sum Sq.** | **Mean Sq.** | **F** | **df** | **p** | **ηp²** |
| --- | --- | --- | --- | --- | --- | --- | --- |
| Negative | Group | 1464.631 | 732.315 | 7.469 | 2, 15 | 0.005** | 0.50 |
|  | Day | 177.73 | 88.865 | 1.756 | 2, 26 | 0.193 | 0.12 |
|  | Group x Day | 1316.013 | 23.848 | 0.471 | 4, 26 | 0.756 | 0.07 |

**Tukey post hoc comparisons**

| **Comparison** | **Diff. of means** | **df** | **q** | **p** |
| --- | --- | --- | --- | --- |
| No shock vs. CS+15 | 14.17 | 3 | 5.354 | < 0.005** |
| No shock vs. CS+3 | 8.058 | 3 | 2.890 | 0.136 |
| CS+3 vs. CS+15 | 6.112 | 3 | 2.492 | 0.216 |

**Positive modulated graded neurons**

**Fig. 8i**

| **Modulation** | **Variable** | **Sum Sq.** | **Mean Sq.** | **F** | **df** | **p** | **ηp²** |
| --- | --- | --- | --- | --- | --- | --- | --- |
| Positive | Group | 0.449 | 0.449 | 29.231 | 1, 15 | < 0.001*** | 0.66 |
|  | Frequency | 0.0709 | 0.977 | 0.977 | 3, 30 | 0.417 | 0.09 |
|  | Group x Frequency | 3.933 | 1.311 | 54.221 | 3, 30 | < 0.001*** | 0.84 |

**Tukey post hoc comparisons**

| **Tone** | **Comparison** | **Diff. of means** | **df** | **q** | **p** |
| --- | --- | --- | --- | --- | --- |
| 3 kHz | CS+3 vs. CS+15 | 0.757 | 2 | 12.335 | < 0.001*** |
| 7 kHz | CS+15 vs. CS+3 | 0.215 | 2 | 3.495 | 0.018* |
| 11 kHz | CS+15 vs. CS+3 | 0.624 | 2 | 10.173 | < 0.001*** |
| 15 kHz | CS+15 vs. CS+3 | 0.702 | 2 | 11.446 | < 0.001*** |

**Fig. 8j**

| **Modulation** | **Variable** | **Sum Sq.** | **Mean Sq.** | **F** | **df** | **p** | **ηp²** |
| --- | --- | --- | --- | --- | --- | --- | --- |
| Positive | Group | 0.055 | 0.055 | 4.795 | 1, 10 | 0.053 | 0.32 |
|  | Frequency | 0.0933 | 0.0467 | 6.548 | 2, 19 | 0.007** | 0.41 |
|  | Group x Frequency | 0.0929 | 0.0464 | 6.518 | 2, 19 | 0.007** | 0.41 |

**Tukey post hoc comparisons**

| **Factor** | **Comparison** | **Diff. of means** | **df** | **q** | **p** |
| --- | --- | --- | --- | --- | --- |
| within CS+15 | 15 vs. 2 | 0.00771 | 3 | 0.228 | 0.986 |
|  | 30 vs. 2 | 0.0194 | 3 | 0.575 | 0.914 |
|  | 30 vs. 15 | 0.0117 | 3 | 0.368 | 0.964 |
| within CS+3 | 2 vs. 15 | 0.236 | 3 | 6.256 | < 0.001*** |
|  | 2 vs. 30 | 0.0346 | 3 | 0.916 | 0.796 |
|  | 30 vs. 15 | 0.202 | 3 | 5.340 | 0.004** |
| within 2 | CS+3 vs. CS+15 | 0.180 | 2 | 4.454 | 0.004** |
| within 15 | CS+15 vs. CS+3 | 0.0639 | 2 | 1.661 | 0.250 |
| within 30 | CS+3 vs. CS+15 | 0.126 | 2 | 3.275 | 0.028* |

**Fig. 8k**

| **Modulation** | **Variable** | **Sum Sq.** | **Mean Sq.** | **F** | **df** | **p** | **ηp²** |
| --- | --- | --- | --- | --- | --- | --- | --- |
| positive | Group | 0.779 | 0.779 | 4.761 | 1, 10 | 0.054 | 0.32 |
|  | Condition | 17.748 | 17.748 | 221.136 | 1, 23 | < 0.001*** | 0.91 |
|  | Group x Condition | 0.671 | 0.671 | 8.366 | 1, 23 | < 0.016* | 0.27 |

**Tukey post hoc comparisons**

| **Factor** | **Comparison** | **Diff. of means** | **df** | **q** | **p** |
| --- | --- | --- | --- | --- | --- |
| within CS+15 | Tone vs. freezing | 2.084 | 2 | 19.458 | < 0.001*** |
| within CS+3 | Tone vs. freezing | 1.405 | 2 | 11.090 | < 0.001*** |
| within freezing | CS+15 vs. CS+3 | 0.0261 | 2 | 0.181 | 0.900 |
| within tone | CS+15 vs. CS+3 | 0.705 | 2 | 4.874 | 0.003** |

**Fig. 8l**

| **Modulation** | **Variable** | **Sum Sq.** | **Mean Sq.** | **F** | **df** | **p** | **ηp²** |
| --- | --- | --- | --- | --- | --- | --- | --- |
| Positive | Group | 5.092 | 5.092 | 0.105 | 1, 10 | 0.752 | 0.01 |
|  | Condition | 17.586 | 8.793 | 0.470 | 2, 19 | 0.632 | 0.05 |
|  | Group x Condition | 20.386 | 10.193 | 0.545 | 2, 19 | 0.588 | 0.05 |

**Negative modulated graded neurons**

**Fig. 8m**

| **Modulation** | **Variable** | **Sum Sq.** | **Mean Sq.** | **F** | **df** | **p** | **ηp²** |
| --- | --- | --- | --- | --- | --- | --- | --- |
| Negative | Group | 0.513 | 0.513 | 13.677 | 1, 10 | 0.004** | 0.58 |
|  | Frequency | 0.00725 | 0.00242 | 0.0836 | 3, 30 | 0.968 | 0.01 |
|  | Group x Frequency | 6.404 | 2.135 | 73.84 | 3, 30 | < 0.001*** | 0.88 |

**Tukey post hoc comparisons**

| **Tone** | **Comparison** | **Diff. of means** | **df** | **q** | **p** |
| --- | --- | --- | --- | --- | --- |
| 3 kHz | CS+15 vs. CS+3 | 1.006 | 2 | 13.781 | < 0.001*** |
| 7 kHz | CS+3 vs. CS+15 | 0.229 | 2 | 3.131 | 0.033* |
| 11 kHz | CS+3 vs. CS+15 | 0.776 | 2 | 10.628 | < 0.001*** |
| 15 kHz | CS+3 vs. CS+15 | 0.841 | 2 | 11.519 | < 0.001*** |

**Fig. 8n**

| **Modulation** | **Variable** | **Sum Sq.** | **Mean Sq.** | **F** | **df** | **p** | **ηp²** |
| --- | --- | --- | --- | --- | --- | --- | --- |
| Negative | Group | 0.00308 | 0.00308 | 0.122 | 1, 10 | 0.733 | 0.01 |
|  | Day | 0.0437 | 0.0218 | 1.323 | 2, 15 | 0.296 | 0.15 |
|  | Group x Day | 0.0299 | 0.015 | 0.907 | 2, 15 | 0.425 | 0.11 |

**Fig. 8o**

| **Modulation** | **Variable** | **Sum Sq.** | **Mean Sq.** | **F** | **df** | **p** | **ηp²** |
| --- | --- | --- | --- | --- | --- | --- | --- |
| Negative | Group | 0.0609 | 0.0609 | 1.421 | 1, 10 | 0.261 | 0.12 |
|  | Day | 3.081 | 3.081 | 443.863 | 1, 10 | < 0.001*** | 0.98 |
|  | Group x Day | 0.0436 | 0.0436 | 6.285 | 2, 15 | 0.031* | 0.46 |

**Tukey post hoc comparisons**

| **Factor** | **Comparison** | **Diff. of means** | **df** | **q** | **p** |
| --- | --- | --- | --- | --- | --- |
| within CS+15 | Tone vs. freezing | 0.813 | 2 | 25.825 | < 0.001*** |
| within CS+3 | Tone vs. freezing | 0.640 | 2 | 17.184 | < 0.001*** |
| within freezing | CS+15 vs. CS+3 | 0.0157 | 2 | 0.240 | 0.868 |
| within tone | CS+15 vs. CS+3 | 0.189 | 2 | 2.888 | 0.062 |

**Fig. 8p**

| **Modulation** | **Variable** | **Sum Sq.** | **Mean Sq.** | **F** | **df** | **p** | **ηp²** |
| --- | --- | --- | --- | --- | --- | --- | --- |
| Negative | Group | 174.242 | 174.242 | 2.001 | 1, 10 | 0.188 | 0.17 |
|  | Day | 11.597 | 5.799 | 0.065 | 2, 19 | 0.937 | 0.01 |
|  | Group x Day | 149.463 | 74.732 | 0.838 | 2, 19 | 0.448 | 0.08 |

**Supplementary File 7 (Fig. 8).** Statistical analysis of positive and negative modulated single-frequency and graded neurons shown in Fig. 8. Two-way ANOVAs with repeated measures were conducted to evaluate effects of group (CS+15, CS+3, or No shock), frequency (3, 7, 11, 15 kHz), day, condition (tone vs. freezing), and their interactions. Tukey post hoc comparisons are shown where simple effects were tested.
