# Supplementary material for: Complementary stable and dynamic prelimbic ensembles encode learned threat value underlying generalization and discrimination
Source: eLife. 2026 Oct 21;15:RP111177. doi: 10.7554/eLife.111177 (PMC13686620; doi:10.7554/eLife.111177)
Supplement: Peer review history

# Editor report: eLife Assessment

**DOI:** 10.7554/eLife.111177.4.sa0

**Authors:** Joshua Johansen

**Affiliations:** RIKEN Center for Brain Science Japan

**Keywords:** Convincing, Valuable

This **valuable** study examines how the rodent prelimbic cortex represents learned and generalized threat over time and identifies distinct stable and dynamic neuronal populations that contribute to these representations. The evidence is **convincing**, supported by longitudinal calcium imaging, appropriate control groups, and sophisticated analyses showing that the relevant neural signals cannot be explained simply by freezing behavior. The work provides a conceptual framework for understanding how stable threat-related representations supporting memory generalization and discrimination can be maintained despite ongoing changes in neuronal ensemble composition.

# Referee report: Reviewer #1 (Public review)

**DOI:** 10.7554/eLife.111177.4.sa1

[Editors' note: this version has been assessed by the Reviewing Editor without further input from the original reviewers. We appreciate the care with which the authors have addressed the remaining concerns. The additional analyses and the revisions to the framing and interpretation have strengthened the manuscript and clarified several of the issues raised during the previous round of review. In particular, the added analyses in Figure 4 and the more precise treatment of repeated retrieval, learned threat value, and the relationship between neural activity and freezing have improved the paper substantially.]

Summary:

The authors combine discriminative auditory fear conditioning with longitudinal in vivo calcium imaging to ask how prelimbic (PL) representations of learned and generalized threat evolve across recent and remote memory time points. Using two different CS+ frequencies and a no-shock control group, they report that PL population activity tracks graded behavioral generalization, that population similarity is highest for tones eliciting strong threat responding, and that distinct subnetworks can be identified that appear to encode tone-specific sensory features versus learned threat-related response structure.

To my knowledge, this may be the first study to comprehensively examine neural encoding of fear generalization in prelimbic cortex (PL). The manuscript is ambitious and technically interesting, and several aspects are potentially important. In particular, the suggestion that neurons showing graded, learning-related response patterns become selectively stabilized over time is intriguing. The inclusion of two CS+ training conditions and a no-shock control also strengthens the case that at least some of the reported effects are related to associative learning rather than simple sensory differences.

# Referee report: Reviewer #3 (Public review)

**DOI:** 10.7554/eLife.111177.4.sa2

Summary:

Normandin et al. explore the coding of stimuli predicting an aversive event in the prelimbic cortex. Stimuli could either be explicitly paired, explicitly unpaired, or novel but with an inferred association with the aversive event (generalization). Long-term tracking of GCaMP positive neurons allowed them to examine how coding evolves out to a month following training. In general, they found two types of ensemble codes. One was ensembles coding for each stimulus independently, but with enhanced responding to the one eliciting a freezing response. The other was ensembles that responded to all stimuli in proportion to their similarity to the stimulus paired with the aversive event, either increasing or decreasing their activation with the degree of freezing elicited by a stimulus. Importantly, this second set of ensembles was more stable across days, potentially providing a memory trace.

Strengths:

(1) The authors track ensembles in prelimbic cortex over long time scales, providing valuable information on the consolidation of neural codes.

(2) Neural coding of generalization is examined, which is under examined in the field.

# Author comment: Author response

**DOI:** 10.7554/eLife.111177.4.sa3

**Authors:** Marc E Normandin, Pedro M Ogallar, Matthew R Lopez, Manuel M Ramos-Alvarez, Isabel A Muzzio

**Affiliations:** University of Iowa Iowa City United States; University of Iowa Iowa City United States; University of Iowa Iowa City United States; Universidad de Jaén Jaén Spain; University of Iowa Iowa City United States

The following is the authors’ response to the previous reviews.

**Public Reviews:**

**Reviewer #1 (Public review):**

Summary:

The authors combine discriminative auditory fear conditioning with longitudinal in vivo calcium imaging to ask how prelimbic (PL) representations of learned and generalized threat evolve across recent and remote memory time points. Using two different CS+ frequencies and a noshock control group, they report that PL population activity tracks graded behavioral generalization, that population similarity is highest for tones eliciting strong threat responding, and that distinct subnetworks can be identified that appear to encode tone-specific sensory features versus learned threat-related response structure.

To my knowledge, this may be the first study to comprehensively examine neural encoding of fear generalization in prelimbic cortex (PL). The manuscript is ambitious and technically interesting, and several aspects are potentially important. In particular, the suggestion that neurons showing graded, learning-related response patterns become selectively stabilized over time is intriguing. The inclusion of two CS+ training conditions and a no-shock control also strengthens the case that at least some of the reported effects are related to associative learning rather than simple sensory differences. However, in its current form, the manuscript does not yet fully support the strength of the conceptual claims. Several issues limit confidence in the interpretation, including the possibility that repeated testing itself contributes to changes across days, uncertainty about the relationship between neural activity and freezing behavior, limited quantitative documentation of longitudinal cell registration, and a number of problems in figure clarity and statistical framing. Overall, the study contains promising observations, but the claims should be narrowed, and several analyses or controls would be needed to fully support the proposed framework.

Comments on revised version.

The authors have addressed my previous concerns well, and the revised manuscript is substantially improved. In particular, the additional analyses strengthen the conclusion that prelimbic cortical activity reflects learned threat value rather than simply freezing behavior, while the revised framing and additional controls clarify the interpretation of the longitudinal neural dynamics. This paper represents an important contribution to our understanding of the neural mechanisms supporting aversive learning, memory, and generalization.

We thank Reviewer 1 for his/her constructive comments, which significantly contributed to strengthen our conclusions.

In response to the reviewer’s recommendations, we added two new analyses to Fig. 4, presented in Fig. 4c–g.

**Reviewer #2 (Public review):**

The authors have substantially revised the paper in response to the original review, which is greatly appreciated. It is clear that it will eventually make a nice contribution to the literature. This being said, the following points are somewhere between major and minor in term of their implications for interpretation of the study results. If they were to be addressed, the paper would be again improved.

We thank Reviewer 2 for the careful second review, which helped us sharpen our arguments and improve the manuscript. The reviewer’s additional comments prompted us to refine the scope and precision of several interpretations. Accordingly, we have removed or clarified potentially confusing language, reframed the longitudinal findings in terms of repeated retrieval rather than the passage of time, and explicitly acknowledged the limitations of relying exclusively on freezing as a behavioral measure. We now also recognize repeated nonreinforced testing as the most direct explanation for the sharpening of the generalization gradients and the responses of newly recruited neurons, while clarifying below how we view this additional learning as contributing to memory updating. We believe these revisions have substantially improved the manuscript, and we are grateful for the reviewer’s thoughtful and detailed feedback.

There are a few remnants of the past language that are not helpful for the interpretation of the study results: (1) "Specifically, the observed population gradients could emerge either from the pooled activity of frequency-selective neurons that respond to individual tones or from neuronal subpopulations that integrate information across tones to encode their learned threat-value."; and (2) "Together, these findings suggest that the PL integrates sensory similarity with learned threat value to generate stable representations that support adaptive generalization and discrimination." Neither of these statement follows what has been shown in the study, even with inclusion of the results from the GLM analysis (see point 4 below).

We directly address this point in response to the reviewer’s point 4

(1) This paragraph in the Discussion is difficult to follow: "Generalization has traditionally been explained by perceptual similarity (Shepard, 1987), whereby stimuli resembling a conditioned cue recruit overlapping sensory representations and evoke similar behavioral responses (Corches et al., 2019; Grosso et al., 2018). Although perceptual similarity clearly influences the extent of generalization, accumulating evidence indicates that it cannot fully account for generalized responding (Verra et al., 2026). More recent frameworks propose that associative learning assigns learned value to novel stimuli by integrating their sensory similarity with previous experience, allowing behavior to scale according to predicted biological significance (Verra et al., 2026; Zaman et al., 2023). Our findings provide a neural framework consistent with these ideas. Sensory similarity promoted consistent neuronal population responses across tones, whereas associative learning organized these responses into graded representations that tracked learned threat value across the stimulus continuum. Thus, sensory similarity appears to define the neuronal substrate upon which associative learning constructs value-based representations that support graded behavioral generalization."

While the revisions have removed the many unnecessary references to inference and integration, this paragraph seems like it is adhering to the original idea of how the authors wished to present their work. If the authors wished to talk about something more than perceptual similarity in the context of generalization, they should have used a task that lends itself to a more-than-perceptual-similarity explanation. Again, the inclusion of the GLM analysis is suggestive for some of what the authors wish to say, but doesn't justify the statements that: "Sensory similarity promoted consistent neuronal population responses across tones, whereas associative learning organized these responses into graded representations that tracked learned threat value across the stimulus continuum." In short, the analysis does not substitute for the design that could have and should have been used to assess learned threat value independently of sensory similarity.

We thank the reviewer for this comment, which helped us identify ambiguity in our terminology. By “learned threat value,” we did not intend to imply that the PL independently infers or computes the value of each novel tone, separate from perceptual similarity. Rather, we used this term to denote the associative significance acquired by the CS+ and CS− during conditioning. Novel cues may then be evaluated according to their perceptual similarity to these learned associations. We have now defined “learned threat value” when it first appears in the manuscript.

Furthermore, we agree that perceptual similarity can account for the graded generalization of responses from the CS+ to neighboring frequencies. We therefore do not consider learned associations and perceptual similarity to be competing explanations. Instead, conditioning determines which stimulus anchors the gradient and its direction according to their acquired “threat value”, whereas perceptual similarity governs how the response extends across intermediate frequencies. This interpretation is supported by our reversed conditioning design: the same frequency continuum produced opposite neural gradients depending on whether 3 or 15 kHz served as the CS+, and no gradients emerged in control animals exposed to the same tones without conditioning. Thus, the physical relationships among the tones shape the graded response, but mnemonic experience determines its organization along the frequency continuum.

The GLM analysis was not intended to demonstrate threat value independently of sensory similarity. Rather, it showed that the learning-dependent gradient remained after accounting for freezing and therefore could not be explained solely by this behavioral response (we address the point of other potential fear responses below).

“Generalization has traditionally been explained in terms of perceptual similarity (Shepard, 1987): stimuli resembling a conditioned cue recruit overlapping sensory representations and therefore evoke similar behavioral responses (Corches et al., 2019; Grosso et al., 2018). More recent frameworks propose that responses to novel stimuli depend not only on sensory similarity but also on mnemonic representations of learned threat value, allowing behavior to scale with the predicted significance of each stimulus (Verra et al., 2026; Zaman et al., 2023). This mnemonic contribution should be especially important when generalization is assessed long after conditioning because responding to a novel cue requires retrieval of the associations formed during learning. Our design did not manipulate sensory similarity and learned threat value independently. However, the reversed conditioning procedure dissociated them in one respect: depending on whether 3 or 15 kHz served as the CS+, the neural gradients ran in opposite directions along the same frequency continuum, with each anchored to the threat-associated tone. A gradient determined by spectral features alone would have run in the same direction in both groups. Thus, the learned associations and their mnemonic representations must have determined the anchor and direction of the neural gradient. By contrast, the contribution of sensory similarity is inferred rather than directly demonstrated: intermediate tones were represented in an order that followed their spectral distance from the CS+. Because discriminative conditioning tends to narrow generalization gradients (Dunsmoor & LaBar, 2013; Herzog et al., 2021; Jenkins & Harrison, 1960; Lommen et al., 2017), tracking neural representations over 30 days allowed us to examine how PL populations evolved as behavioral discrimination became progressively more precise during repeated retrieval. Whereas stable neuronal populations retained consistent response profiles across retrieval sessions, neurons recruited after conditioning exhibited changes that paralleled the behavioral sharpening. These results suggest that memory, new learning, and sensory similarity interact to shape generalization gradients.”

(2) The next paragraph in the Discussion is also confusing. "Such reorganization has been proposed to provide flexibility by allowing new information to be incorporated into existing cortical representations while preserving stable behavioral performance (Mau et al., 2020; Zaki & Cai, 2024). Several mechanisms could contribute to this turnover, including systems consolidation, retrieval-induced reconsolidation or memory updating, and repeated nonreinforced stimulus exposure (Lacagnina et al., 2019; Mau et al., 2020; Sangha, 2015; Zaki & Cai, 2024). Although our experiments cannot distinguish between the first two possibilities, the behavioral data argue against extinction as the primary explanation. Extinction is generally associated with the formation of new CS+-safety associations (Bouton et al., 2021), whereas discrimination ratios increased across retrieval sessions, indicating that animals progressively improved their discrimination between threat-associated and safe stimuli rather than acquiring generalized safety responses. This pattern is consistent with previous work showing that discrimination learning sharpens stimulus representations and narrows behavioral generalization gradients (Dunsmoor & LaBar, 2013; Herzog et al., 2021; Jenkins & Harrison, 1960; Lommen et al., 2017). Importantly, turnover was not uniform across the population. Graded neurons retained remarkably consistent response profiles across retrieval sessions, and their activity remained more strongly associated with learned threat value than with freezing behavior. These observations indicate that stable components of the population code can coexist with extensive reorganization of surrounding neuronal ensembles."

The issue with repeated testing is not caused by extinction per se. The issue is that nonreinforcement across the repeated testing should differentially affect the CS+ and CS-. Specifically, it should extinguish responding to the CS- stimulus at a rate that matches its distance from the CS+, thereby sharpening the CS+ versus CS- discrimination in precisely the ways that have been observed. Ergo, the repeated testing is a problem for inferences that might be drawn about the way that generalization gradients change with; and is a problem for statements regarding "dynamic reorganization of cortical activity patterns over time." There is nothing in the study that allows one to comment on the reorganization of cortical activity patterns over time. The reorganization can and should be attributed to the repeated testing, which is confounded with time. Nonetheless, the reorganization must be due to the repeated testing and NOT time as the present findings are inconsistent with the well-documented broadening of generalization gradients with time.

We agree with the reviewer that discrimination between the CS− and CS+ sharpened across retrieval sessions and that repeated testing may have contributed to this effect. Our discussion of extinction was included specifically to address a concern previously raised by Reviewer 1 and to clarify that the observed sharpening did not reflect a uniform reduction in conditioned responding. We now only left this explanation in the results section.

We also agree that later retrieval sessions are not passive readouts of the original memory. Repeated presentation can provide new opportunities for learning, update reactivated memory traces, and initiate reconsolidation. The intermediate tones illustrate this point particularly well: they are novel during the first retrieval session but become familiar through subsequent exposure, and their representations may therefore change across sessions. Thus, new learning, memory updating, and reconsolidation are not mutually exclusive explanations but interacting processes engaged by repeated retrieval. We have revised the Discussion to acknowledge these contributions more explicitly.

We also agree that our use of phrases such as “over time” was imprecise. We have replaced this language with “across retrieval sessions” to avoid implying that the passage of time alone produced ensemble turnover.

However, we respectfully disagree that cortical reorganization cannot be evaluated across repeated retrieval sessions or that repeated testing alone necessarily accounts for all the observed changes. Multiple studies have documented turnover within cortical ensembles following learning (Lacagnina et al., 2019; Mau et al., 2020; Sangha, 2015; Zaki & Cai, 2024). Importantly, we observed ensemble turnover by test day 1, before repeated testing could have exerted a cumulative effect, and similar early turnover has been reported by other laboratories (Kitamura et al., 2017; DeNardo et al., 2019). These previous studies have also interpreted changes in ensemble composition between conditioning and subsequent retrieval sessions, including remote retrieval, as evidence of cortical reorganization. Our paradigm differs from these studies because we introduced novel tones to examine how representations of the original CS+/CS− associations interact with representations of perceptually similar cues.

In summary, we agree with the reviewer that repeated testing may generate additional learning that contributes to the sharpening of behavioral generalization gradients and the responses of newly recruited neurons. However, we view this learning as inseparable from memory updating and reconsolidation, because information acquired during retrieval must be incorporated into existing memory representations to persist across sessions.

“The ensemble turnover observed here is consistent with previous studies demonstrating dynamic reorganization of cortical activity patterns across temporally separated retrieval sessions (DeNardo et al., 2019; Gallego et al., 2020; Kitamura et al., 2017; Tome et al., 2024). Such reorganization has been proposed to provide flexibility by allowing new information to be incorporated into existing cortical representations while preserving stable behavioral performance (Mau et al., 2020; Zaki & Cai, 2024). Several processes could contribute to this turnover, including additional learning during repeated nonreinforced testing, memory updating, and reconsolidation (Lacagnina et al., 2019; Mau et al., 2020; Sangha, 2015; Zaki & Cai, 2024). In our experiments, turnover was already evident at the first retrieval session, before repeated testing could have exerted a cumulative effect, indicating that this reorganization emerges early after learning. Nevertheless, repeated testing provides a plausible explanation for changes during subsequent sessions. Each retrieval session reactivated the original CS+/CS− associations while presenting the conditioned and intermediate tones without reinforcement, thereby creating opportunities for additional discrimination learning. These experiences may have progressively sharpened differentiation between the CS+ and CS-, modifying the retrieved memory representations and changing which neurons participated in representing the refined associations. Although our data do not establish a mechanistic link between neuronal turnover and additional learning, they raise the possibility that dynamic ensemble membership provides the flexibility needed to incorporate new information.”

(3) In the next paragraph, the authors state: "At the same time, narrower generalization gradients and improved discrimination across retrieval sessions suggests ongoing memory updating. These observations are consistent with contemporary theories proposing that systems consolidation and retrieval-dependent updating are complementary processes through which memories continue to evolve after learning (Mau et al., 2020; Tome et al., 2024; Zaki & Cai, 2024)."

In general, I'm not sure why one would invoke systems consolidation or retrieval-induced reconsolidation as an explanation for any of the present findings: they are not explanations of much at all. In this specific text, the authors seem to be implying an updating process that occurs independently of what is learned across the repeated sessions of testing. Why? The changes that occur in the behaviour and neuronal representations are perfectly explicable in terms of additional learning that occurs - of the sort that I hope to have made clear in my previous comment. Why invoke more than what is needed to explain the observed pattern of results?

We appreciate the reviewer’s clarification. As discussed in our response to the preceding comment, we agree that repeated nonreinforced testing provided additional learning opportunities and offers the most direct explanation for the progressive sharpening of behavioral discrimination and neuronal responses. We recognize that our original wording could have been interpreted as proposing memory updating as a process occurring independently of learning during the test sessions. This was not our intention. By “memory updating,” we meant that new information acquired during repeated retrieval modified the existing representations of the CS+ and CS−, allowing these stimuli to become more clearly differentiated. Thus, the additional learning that occurs through repeated testing provides the experience through which the original associations are refined.

We agree that systems consolidation and retrieval-induced reconsolidation are not required as direct explanations for the observed sharpening and that our design cannot isolate their contributions. However, ensemble turnover was evident by day 1, before repeated testing could have exerted cumulative effects, indicating that repeated testing cannot fully explain the turnover. We view the additional learning acquired during subsequent retrieval sessions as refining the represented values of the CS+ and CS− and, if these changes persist, as requiring incorporation into existing memory representations through updating and reconsolidation. These processes therefore complement, rather than compete with, additional learning. The revised Discussion distinguishes repeated testing as the most direct explanation for the sharpening of discrimination from the broader memory processes that may accompany this learning and contribute to cortical ensemble reorganization.

“In summary, PL population responses formed generalization gradients that paralleled behavioral generalization and were primarily organized according to tone-learned threat value. The overlap among responses to conditioned and novel tones was consistent with their perceptual similarity, whereas repeated nonreinforced testing was accompanied by progressive sharpening of both behavioral discrimination and the response gradients expressed by neurons recruited after conditioning. Together, these findings provide a potential neural framework through which stored threat associations may guide the evaluation of perceptually similar cues.”

“The coexistence of these stable neurons with dynamic ensembles may allow repeated retrieval to refine a memory while preserving core features of its original representation (Mau et al., 2020; Tome et al., 2024; Zaki & Cai, 2024). Determining whether these stable graded neurons are causally required for memory storage or retrieval—and whether they constitute a persistent cortical memory trace—will require longitudinal imaging combined with selective manipulation of this population.”

(4) Re the GLM analysis - The authors write that: "the fact that the GLM analysis indicates that these neurons reflect learned threat value more than freezing behavior, suggests that they encode an abstract property of the learned stimulus rather than simply mirroring behavioral output."

This is fine if freezing fully indexes the state of conditioned fear and there are no other behaviours in which animals express their fear. If, however, fear is expressed in a range of other behaviours that are likely coordinated by the PL (e.g., startle, vigilance, scanning, orienting to source of danger), this interpretation of the GLM analysis is unwarranted. This is an important point and would be worth noting somewhere in the paragraph where the statement appears.

We agree with the reviewer. Our GLM demonstrates that the graded neuronal responses cannot be explained by freezing behavior alone, but it does not exclude contributions from other unmeasured manifestations of conditioned fear, including vigilance, scanning, orienting, startle, or physiological responses. We have therefore removed the claim that these neurons primarily encode threat value and revised the Discussion to acknowledge this limitation explicitly.

“Freezing, however, does not exhaust the conditioned defensive state, and we cannot rule out contributions from unmeasured behavioral or physiological correlates of fear, including vigilance, scanning, orienting, startle, and changes in autonomic state. Our findings are therefore consistent with PL neurons representing the learned significance of the stimuli, but they do not establish that this representation is independent of all fear-related behavioral and physiological states.”

**Reviewer #3 (Public review):**

Summary:

Normandin et al. explore the coding of stimuli predicting an aversive event in the prelimbic cortex. Stimuli could either be explicitly paired, explicitly unpaired, or novel but with an inferred association with the aversive event (generalization). Long-term tracking of GCaMP positive neurons allowed them to examine how coding evolves out to a month following training. In general, they found two types of ensemble codes. One was ensembles coding for each stimulus independently, but with enhanced responding to the one eliciting a freezing response. The other was ensembles that responded to all stimuli in proportion to their similarity to the stimulus paired with the aversive event, either increasing or decreasing their activation with the degree of freezing elicited by a stimulus. Importantly, this second set of ensembles was more stable across days, potentially providing a memory trace.

Strengths:

(1) The authors track ensembles in prelimbic cortex over long time scales, providing valuable information on the consolidation of neural codes.

(2) Neural coding of generalization is examined, which is under examined in the field.

Comments on revised version.

The authors have convincingly and thoroughly addressed my concerns. I have no further issues regarding this study.

We thank reviewer 3 for his thoughtful and constructive comments and the suggestion to use the GLM which greatly improved the interpretation of our data.

**Recommendations for the authors:**

**Reviewer #1 (Recommendations for the authors):**

One small point is that the authors could report the beta coefficients for the tone and freezing predictors from the GLM analysis shown in Figure 4. Providing these values would make it easier to directly compare the relative contributions of tone-related and freezing-related activity to the model.

We now include this analysis in Figure 4c and 4g
